# Ecological study of the association between socioeconomic inequality and food deserts and swamps around schools in Rio de Janeiro, Brazil

**DOI:** 10.1186/s12889-023-14990-8

**Published:** 2023-01-17

**Authors:** Bernardo Andretti, Letícia Oliveira Cardoso, Olivia Souza Honório, Paulo César Pereira de Castro Junior, Letícia Ferreira Tavares, Isabela da Costa Gaspar da Silva, Larissa Loures Mendes

**Affiliations:** 1 Brazilian School of Public and Business Administration, Getulio Vargas Foundation, 30 R. Jornalista Orlando Dantas, Rio de Janeiro, CEP 22231-010 Brazil; 2 Sergio Arouca National School of Public Health, Oswaldo Cruz Foundation, Rio de Janeiro, Brazil; 3 Nutrition School, Federal University of Ouro Preto, Ouro Preto, Brazil; 4 Institute of Nutrition Josué de Castro, Federal University of Rio de Janeiro, Rio de Janeiro, Brazil; 5 Nutrition Department, Federal University of Minas Gerais, Belo Horizonte, Minas Gerais Brazil

**Keywords:** Community food environment, Spatial neighborhood inequalities, Food deserts, Food swamps, Schools

## Abstract

**Background:**

Previous research suggests that unhealthy community food environments around schools contribute to unhealthy eating habits and negative health outcomes among the youth. However, little is known about how socioeconomic inequalities in those community food environments are associated with food deserts and food swamps across schools’ neighborhoods.

**Methods:**

An ecological study was carried out in all 3,159 public and private schools in Rio de Janeiro, Brazil. Three measures of socioeconomic inequality were evaluated: per capita income, segregation index and deprivation index. The community school food environment was analyzed by metrics of food swamps and food deserts.

**Results:**

Food deserts and food swamps were simultaneously more prevalent in neighborhoods of the lowest income, high deprivation, and high segregation. Spatial socioeconomic disparities at the neighborhoods of schools were associated with food deserts and food swamps in Rio de Janeiro.

**Conclusions:**

Our results point to a spatial socioeconomic inequality of establishments that sell food around schools in a Brazilian metropolis, indicating that the areas of greatest deprivation of food services are also the areas with the worst socioeconomic characteristics.

**Supplementary Information:**

The online version contains supplementary material available at 10.1186/s12889-023-14990-8.

## Introduction

The community food environment has been conceptualized as an important driver of individuals’ eating patterns [1]. This is also true for the school settings in cities, which are placed inside communities – or neighborhoods – with access to healthy and plentiful unhealthy food outlets. School vicinities are places where social interactions and commuting take place [2]. Moreover, they are usually located in neighborhoods characterized by both food deserts and food swamps, which are correlated with obesity [3] and unhealthy eating behaviors among the youth [4–7].

Socioeconomic and spatial disparities of the food environments, in turn, are associated with dietary choices [8] and racial segregation [7], with direct impact on the youth health status [5, 9]. Specifically, neighborhood school food environments in low-and-middle-income countries (LMIC) predominantly retail unhealthy foods, which contribute to unhealthy eating habits among the youth [10–14]. Thus, shedding light into how inequalities play a role in shaping the availability of healthful food choices in the community school food environment [15] is crucial to further deepen our understanding of the so-called nutritional inequality [16–18], which not only influences food behaviors of children and adolescents, but also helps shaping their eating habits throughout life. Further, while most studies describing socioeconomic disparities at the school or the community around the school food environments are focused on the US and other high-income countries [14, 19], we also add to the body of research by extending the findings to the LMIC context.

We thus analyze how different measures of inequalities at the neighbourhood level – measured by *per capita* income, public vs. private schools, and indices of spatial segregation and deprivation – are associated with disparities in food availability in the community food environment around schools in Rio de Janeiro, Brazil. Finally, we investigate the prevalence of food deserts and food swamps, stratified by each measure of spatial inequality.

## Method

### Study sample

We conducted an ecological study across all public and private schools of the second largest city in Brazil. Rio de Janeiro has 6,775.561 inhabitants and a Human Development Index (HDI) of 0.799. While 31.4% of its citizens have a per capita income of half a minimum wage, the average per capita income is around 4.2 minimum wages.

Secondary database from 2019 was extracted from the State Education Secretariat (*Secretaria Estadual de Educação –* SEE). Seventy-nine schools were excluded from the final sample. 38 offered only professional education, 7 that attended only special education, and 34 with missing segregation data. The final sample comprised 3,159 schools, which are categorized into public and private.

### Neighborhood inequality measures

We collected three measures of socioeconomic spatial inequality, and aggregated at the neighborhood level—which is composed of an aggregate of census tracts: (i) *per capita* income, (ii) segregation index, and (iii) deprivation index. Data to build the indicators was collected from the last 2010 country census. Besides, we converted all current values from Brazilian Real to US Dollars, using 2010 as the conversion date. Importantly, census tracts are defined in Brazil as the smallest territorial unit, formed by a continuous area, fully contained in an urban or rural area and determined according to the number of households [20]. 

Per capita income was calculated as a ratio between total income of the neighborhood and its population. We categorized *per capita* income into terciles: lowest tercile ranging from U$249.21 and U$593.43; Middle tercile from U$593.44 and U$1,022.44; and highest tercile from U$1,022.55 and U$6,510.32.

As per the segregation index, we used the Getis-Ord Local Gi*statistic (or Gi*statistic). It encompasses a spatially weighted Z-score that represents how much a neighborhood’s income composition deviates from the larger metropolitan areas in its surroundings. Therefore, this index is unique because it (a) takes into account the spatial clustering of segregation within cities, and (b) considers racial composition and social inequalities within and between neighborhoods.

The segregation index is calculated as the standard deviations (SD) between the economic composition of the neighborhoods—assessed by the proportion of heads of household in neighborhoods that earn a monthly income within 0 to 3 SD of the minimum wage—in relation to the surrounding neighborhoods. Thus, we can detect segregation at neighborhood-level and thereby examine segregation within metropolitan areas. Data from the 2010 Brazilian Census were used to determine the proportion of heads of households in a neighborhoods earning a monthly income within 0–3 minimum wage (approximately US$ 0·00–US$ 900·00 in 2010) [21].

The census tracts were weighted using a first-order rook spatial weight matrix and three categories of segregation were created: (1) High: Gi * statistic ≥ 1·96; (2) Medium: Gi * statistic between 0 and 1·96 and (3) Low: Gi * statistic < 0 according to the distribution of the Z-score. Higher, positive scores represented census tracts that are more segregated—meaning that the proportion of households receiving 0–3 minimum wages are overrepresented in the neighborhood, while lower, negative scores suggest the opposite. Values close to 0 represent neighborhoods in which spatial segregation is low.

Finally, the deprivation index was retrieved from a recently published technical report [22]. It is calculated as a combination of three main indicators: (i) percentage of households receiving less than ½ minimum wage, (ii) percentage of illiterate inhabitants age 7 or older, and (iii) average of individuals with inadequate access to sanitation. Because this index is available for census tracts and our unit of analysis is the neighborhood, we average those sectors and aggregate their value to fit the neighborhood unit of analysis. We then use as thresholds the average of deprivation +-½ SD. Low deprivation areas are those below the lower end of the average vulnerability subtracted by ½ SD, while high deprivation is above the average + ½ SD. Medium deprivation is the interval between the SD from the average. Then, we also categorized this index into three, based on the Health Vulnerability Index [23]: high, medium, and low deprivation neighborhoods.

### Community school food environment

Secondary database of food establishments from 2019 was extracted from the Rio de Janeiro State Treasure Secretariat (*Secretaria Estadual da Fazenda*). We classified establishments as food stores based on the National Classification of Economic Activities (*Classificação Nacional de Atividades Econômicas,* CNAE^1^), which informs the main economic activity of each registered establishment.

Food establishments were classified in accordance with the Technical Study on Mapping Food Deserts in Brazil—CAISAN), in which categorizes establishments in three: (i) in natura establishments, which included butcheries, seafood shops, and fruits and vegetables establishments; (ii) ultraprocessed establishments, which included bars, snack bars, convenience stores, candy shops, and street vendors; and (iii) mixed establishments, which included markets, hypermarkets, mini markets, bakeries, restaurants and food stores (general food stores as natural and dietetic products, frozen foods, ice-cream stores, cake factories, warehouses, and commercial food stores with predominant retail of processed foods) [24].

Furthermore, the food environment around the schools was analyzed considering food deserts and swamps. Food deserts are defined as neighborhoods with limited access to healthy food, while food swamps are defined as neighborhoods with high availability of unhealthy foods [25]. To determine food deserts,  we adopted the methodology proposed by CAISAN, which calculates the density of healthy establishments per 10,000 inhabitants. Healthy establishments correspond to the sum of in natura acquisition and mixed establishments. Given the above, food deserts are the neighborhoods that are below the percentile for the distribution of the density of healthy establishments [24]. In the present study, the 25th percentile corresponds to 27.35. To determine food swamps, on the other hand, we calculated the sum of convenience stores, snack bars, grocery stores and candy stores. When the sum of these establishments in the neighborhood was greater than four establishments, the neighborhood was classified as a food swamp [5, 6]. We thus used the adapted metric of Hager and colleagues (2017), first utilized by Peres and colleagues (2017). In this adapted metric, we account for the Brazilian context by adding Cafeterias (‘*lanchonetes’*)—food establishments largely characterized by the abundance of ultraprocessed foods. Additional results using mRFEI can be found in the Appendix.

### Statistical analysis

Descriptive statistics are presented in absolute and relative frequencies. We calculated measures of central tendency as the median and  the interquartile range (p25-p75). We compared relative frequencies using chi-square tests. The analyses were performed in SPSS 19.0, and maps were elaborated in QGis 2.14.9.

## Results

**Fig. 1:**
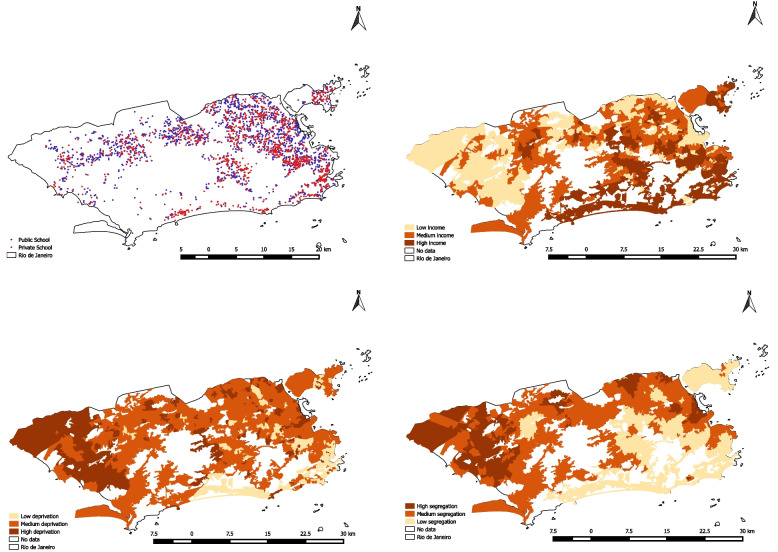
Spatial Distribution of Public and Private Schools and Socioeconomic Indicators of the city of Rio de Janeiro **A**: Public and private schools; **B**: Tercile of neighborhood *per capita* income; **C**: Deprivation Index; **D**: Segregation Index

Figure 1 presents the distribution of public and private schools in the city of Rio de Janeiro, as well as the neighborhood spatial distributions of the inequality measures. From a visual inspection, we can observe that the wealthier and less segregated area is located in the south-east region, while the poorer and more segregated area is in the north-west of the city. However, we can also observe that there are regions of the city in which wealthy and poor regions nearly coexist.

Summary statistics of the distribution of schools and the three inequality measures are presented in Table 1. Most schools are public (55.0%), and located in neighborhoods of the highest income bracket (40.2%), medium deprivation index (64.1%) and medium segregation (44.7%).

**Table 1:** Summary statistics of schools and neighborhood inequality measures in Rio de Janeiro

	n	%
**Type of School**		
Public	1737	55.0
Private	1422	45.0
***Per capita* Income**		
Lowest tercile	753	23.9
Middle tercile	1135	35.9
Highest tercile	1271	40.2
**Deprivation Index**		
High Risk	538	17.0
Medium Risk	2025	64.1
Low Risk	596	18.9
**Segregation Index**		
High (≤ 1,96)	390	12.3
Medium (0 to 1,96)	1412	44.7
Low (< 0)	1357	43.0

**Fig. 2:**
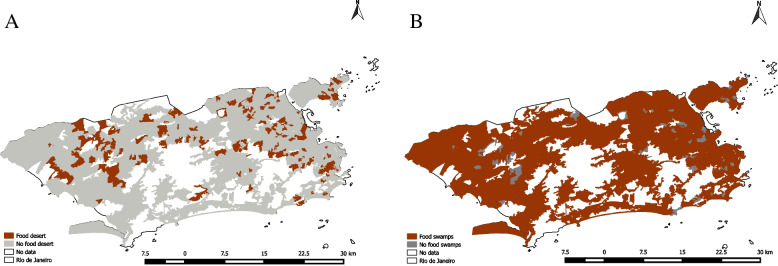
Distribution of Food Deserts and Food Swamps **A**: Food Deserts; **B**: Food Swamps

Figure 2 displays the spatial distribution of food deserts and food swamps. Visually, we observe that there is a high prevalence of food swamps across neighborhoods of the city of Rio de Janeiro, but not quite as many food deserts.

Table 2 presents descriptive statistics of the neighborhood community school food environment. Most schools have at least one food establishment in its neighborhood (99.7%), in which 98.5% had a presence of at least one ultraprocessed establishment and 93.3% at least one *in natura* establishment. Across all establishment classifications, private schools have more establishments in their neighborhoods considering median values and interquartile ranges. While public schools had in their neighborhood a median of one *in natura* establishments, private ones had two. The same was true for the median of ultraprocessed (6 vs. 13) and mixed (10 vs. 17) establishments in public vs. private schools. Results from Table 2 also reveal a higher prevalence of establishments selling either ultraprocessed or mixed foods, when compared to predominantly *in natura* establishments.

**Table 2:** Distribution of food establishments at the neighborhood school food environment in Rio de Janeiro, 2019

	**Schools with at least one establishment**	**Schools—Total**	**Private Schools**	**Public Schools**
	% (n)	Median (Interquartile Range)	Median (Interquartile Range)	Median (Interquartile Range)
**Predominantly *In natura* Establishments**	**93.3 (2948)**	**9 (3–21)**	**2 (0–5)**	**1 (0–4)**
Butcheries	88.7 (2802)	5 (2–11)	1 (0–3)	1 (0–3)
Fruit and Vegetable market	75.0 (2370)	3 (1–8)	0 (0–2)	0 (0–1)
Seafood market	47.1 (1487)	0 (0–2)	0 (0–0)	0 (0–0)
**Predominantly Ultraprocessed Establishments**	**98.5 (3111)**	**44 (15–155)**	**13 (4–32)**	**6 (2–17)**
Street vendors	72.3 (2283)	2 (0–18)	0 (0–2)	0 (0–1)
Bars	81.8 (2583)	4 (1–16)	1 (0–4)	0 (0–2)
Cafeterias	97.9 (3092)	32 (11–91)	9 (3–23)	5 (1–13)
Convenience stores	29.7 (937)	0 (0–1)	0 (0–0)	0 (0–0)
Candy shops	78.4 (2477)	3 (1–9)	1(0–2)	0 (0–1)
**Mixed Establishments**	**99.6 (3145)**	**61 (29–150)**	**17 (7–37)**	**10 (4–23)**
Hypermarkets and supermarkets	94.4 (2983)	10 (4–21)	3 (1–5)	2 (0–5)
Food stores*	90.6 (2862)	7 (2–21)	2 (0–5)	1 (0–3)
Mini markets	97.7 (3087)	16(8–36)	4 (1–8)	3 (1–7)
Bakeries	92.2 (2913)	6 (3–16)	1 (0–3)	1 (0–3)
Restaurants	94.4 (2982)	17 (5–61)	5 (1–16)	2 (0–6)
**Total**	**99.7 (3149)**	**119 (50–330)**	**33 (13–73)**	**20 (7–45)**

^*^These establishments encompass general food stores. This category includes, but is not limited to, natural and dietetic products, frozen foods, ice-cream stores, cake factories, warehouses, and commercial food stores with predominant retail of processed foods

Table 3 displays the presence of food deserts, food swamps, and both simultaneously at the school neighborhood community food environments. Overall, 474 (15%) of schools were in neighborhoods categorized as food deserts, 3094 (97%) were in neighborhoods categorized as food swamps, and 380 (12%) were in neighborhoods categorized by both food deserts and swamps simultaneously. Food deserts are more prevalent in neighborhoods of public vs. private schools (17.0 vs. 12.6%, *p* = 0.001); in neighborhoods of the lowest income tercile (34.5%) vs. middle tercile (9.2%) and highest tercile (8.7%, *p* < 0.001); in neighborhoods of high deprivation (32.7%) vs. medium (11.6%) and low (10.7%, *p* < 0.001); and in neighborhoods of high segregation (34.1%), vs. medium (12.6%) and low (12.0, *p* < 0.001).Food swamps, on the other hand, are much more prevalent overall, showing no statistical difference on the deprivation index (*p* = 0.231), and only a marginal difference on the  segregation index (*p* = 0.079). However, food swamps are slightly more prevalent in private when compared to public schools (98.3% vs. 95.9%, *p* < 0.001), and in the middle and highest income terciles, compared to the first (99.6%, 99.1%, and 89.6%, respectively, *p* = 0.016).When assessing both food deserts and food swamps combined, we observe a similar pattern of results as in the food deserts. While we observe no significant differences in public and private schools (12.9% vs. 11%, *p* = 0.098), food deserts and food swamps are simultaneously more prevalent in neighborhoods of the lowest income tercile (24.2%) vs. middle tercile (8.7%) and highest tercile (7.8%, *p* < 0.001); in neighborhoods of high deprivation (25.7%) vs. medium (9.0%) and low (10.1%, *p* < 0.001); and in neighborhoods of high segregation (24.4%), vs. medium (9.7%) and low (10.9, *p* < 0.001).

**Table 3:** Food deserts and food swamps in the neighborhood of schools in Rio de Janeiro

		**Food Deserts**			**Food Swamps**		**Food Deserts and Swamps**			
		*n*	%	*p*-value*	*n*	%	*p*-value*	*n*	%	*p*-value*
**Type of School**										
Public	**1737**	295	17.0	**0.001**	1666	95.9	** < 0.001**	224	12.9	0.098
Private	**1422**	179	12.6		1398	98.3		156	11.0	
***Per capita* Income**										
Lowest tercile	**753**	260	34.5	** < 0.001**	675	89.6	**0.016**	182	24.2	** < 0.001**
Middle tercile	**1135**	104	9.2		1130	99.6		99	8.7	
Highest tercile	**1271**	110	8.7		1259	99.1		99	7.8	
**Deprivation Index**										
High	**538**	176	32.7	** < 0.001**	499	92.8	0.231	138	25.7	** < 0.001**
Medium	**2025**	234	11.6		1973	97.4		182	9.0	
Low	**596**	64	10.7		592	99.3		60	10.1	
**Segregation Index**										
High	**390**	133	34.1	** < 0.001**	352	90.3	0.079	95	24.4	** < 0.001**
Medium	**1412**	178	12.6		1371	97.1		137	9.7	
Low	**1357**	163	12.0		1341	98.8		148	10.9	
**Total**	**3159**	**474**	**15.0**		**3064**	**97.0**		**380**	**12.0**	

^*^P-values were retrieved from the comparison of relative frequencies using chi-square tests

## Discussion

Our study documents that the spatial socioeconomic disparities across neighborhoods of schools in Rio de Janeiro—measured by income terciles, and indices of segregation and deprivation – are associated with higher prevalence of both food deserts and food swamps. We find that food deserts and swamps are simultaneously more present in school’s neighborhoods of lower-income, higher deprivation, and higher segregation. This result offers additional evidence that spatial socioeconomic inequalities may influence school food environments, which thus far has not been a consensus in the literature [14, 16–18], which has mostly focused on high-income countries [14, 19].

Our contributions to the literature on spatial inequalities and community school food environments are twofold. First, we assess a unique context of LMIC countries with higher levels of inequality—which is the case of Brazil in general, and Rio de Janeiro in particular, a large city that is famous for its near coexistence of slum conglomerates and wealthy neighborhoods. Second, by utilizing novel measures of spatial inequality, we seek to help the development and the validation of new indices and propose a new lens to analyze the spatial inequalities within cities, and how they may be associated with food environments around schools.

While some studies show that more deprived schools display a higher prevalence of overall food outlets [26] and fast-food outlets [27] compared to higher-income ones in the US, others document the opposite in schools of New Zealand [28]. In another Brazilian metropolis, Belo Horizonte, higher-income schools displayed a higher prevalence of all food stores (including restaurants, bars, and snack bars) in their vicinity, except for markets and supermarkets [6]. Interestingly, having access to convenience stores with higher availability of energy-dense and nutrient-poor foods was frequently associated with overweight and obesity in Hispanic and Black youth. A systematic review shows that the higher presence of fast food outlets near US schools was associated with a higher prevalence of obesity among Latino, Black, and White students, with mixed results for Asian students [19]. Furthermore, a recent systematic review presents mixed results [29] by documenting only few significant associations between food environment features and health outcomes.

In a cross-sectional study that examined the New York City food environment around homes and public schools, stratified by race/ethnicity and poverty status, low-income Black, Hispanic, and Asian students lived and attended schools located closer to nearly all food outlets (including corner stores, fast food outlets, wait service restaurants, and supermarkets), when compared to low-income White students [30], which implies that low-income non-white children were more likely to both live and study nearer food stores. In a sample of all public schools in the US, Hispanic students were also more likely to attend schools surrounded by convenience stores, restaurants, snack stores, and off-licenses [31]. Using national data from public schools in the US, another study shows that students in higher poverty-level schools and those with majority Black and majority Hispanic students had lower access to unhealthy food outlets than students in higher income, majority White, and diverse schools. The results further suggest that high poverty and black and hispanic majority schools tend to be exposed to healthier school food environments than other types of school, even though nutritional quality of meals offered did not significantly differ between groups [32]. Although we do not directly assess racial inequalities in our study, in Rio de Janeiro (and in Brazil) the most deprived areas are also those with higher proportions of Black residents.

Finally, our study is closely related to previous findings from Mexico [33] and Spain [34]. In both studies and ours, we can observe that the amplification of deprivation and segregation lead to lower and poorer access to services and goods in the surroundings. In the context of three Mexican cities, school food environments of regions with higher poverty and lower educational attainment were associated with higher availability of ultraprocessed foods and beverages [33]. In Madrid, schools located in poorer neighborhoods presented a higher density of unhealthy food establishments in their vicinity [34]. In our study, we find that neighborhoods of lower-income, and highly deprived and segregated schools are more likely to present both food deserts and food swamps.

We provide novel evidence of a different set of inequalities mostly focused on income, spatial inequalities, and access to basic services. We show a consistency of prevalence in food deserts and swamps when considering all inequality measures: income, segregation, and deprivation. Taken together, our findings suggest that socioeconomic and spatial inequality measures can foster the understanding of the community food environments. Further research, however, is required to address mechanisms and causal links through longitudinal analyses, and to test the effectiveness of policies aiming at reducing such food availability inequalities. This would fill a gap that this study leaves as limitations. Though our study offers several insights, it provides correlational evidence of the association between socioeconomic indices and the community food environment around schools.

As an example of public policies tailored to improve the community food environment, member States of the World Health Organization (WHO) approved in 2010 a package of recommendations regarding targeted commercialization of food and non-alcoholic beverages to children and adolescents, including specific restrictions to the exposure of publicity of such foods and beverages at the school food environment—which comprises all spaces and facilities in and around schools where food and beverages are available to be sold and purchased [35]. It is worth noting that Brazil is a reference country in creating effective policies that help ensure the supply of a healthy and adequate diet inside the public schools, mainly by the implementation of the National School Feeding Program [36], but not as much in private ones [37].

Further, the literature highlights the need to improve not only food environments inside schools, but also in its vicinities. For instance, modifying zoning regulations that restrict access to fast-food outlets around schools has been suggested as an effective policy to reduce unhealthy eating among school children in Quebec [13]. More recently, the Food and Agriculture Organization of the United Nations (FAO) published a guide to develop law proposals targeting improvements of food security and nutrition at the school food environments, including their neighborhood’s food environments. The most relevant recommendations include urban public planning and new legislation that regulates commercial licensing that defines incentives and rules for a healthy retail food system, both inside and at the school’s vicinity [38]. Importantly, by increasing access and availability of healthy products, such regulation may ensure (or at least contribute to) a healthier and more adequate diet for children and adolescents [1, 10–12, 14].

Therefore, our results, in line with recent UN guidelines, suggest that policy implications may involve increasing regulation in food environments across school’s neighborhoods and, finally, creating targeted and effective programs to reduce nutritional inequality across cities.

## Supplementary Information

**Additional file 1.**

## Data Availability

The datasets used and/or analyzed during the current study are available from the corresponding author on reasonable request. All original data used is public. Data on schools are available at https://inepdata.inep.gov.br/analytics/saw.dll?dashboard. Data on neighborhood inequality measures are available at https://censo2010.ibge.gov.br/resultados.html, and on Deprivation index available at: https://researchdata.gla.ac.uk/980/. Data on the community food environment of Rio de Janeiro is not opened to the public, but available upon request at https://www.seeduc.rj.gov.br.

## Footnotes

1 https://concla.ibge.gov.br/classificacoes/por-tema/atividades-economicas/classificacao-nacional-de-atividades-economicas.html
