# Supplementary material for: Ecological study of the association between socioeconomic inequality and food deserts and swamps around schools in Rio de Janeiro, Brazil
Source: BMC Public Health. 2023 Jan 17;23:120. doi: 10.1186/s12889-023-14990-8 (PMC9847189; doi:10.1186/s12889-023-14990-8)
Supplement: Supplementary file 1 — Additional file 1. [file 12889_2023_14990_MOESM1_ESM.docx]

| Food deserts and food swamps around schools of Rio de Janeiro using mRFEI. | | | | | | |
| --- | --- | --- | --- | --- | --- | --- |
|  | **Food Deserts** | | | **Food Swamps** | | |
|  | n | % | p | n | % | p |
| **Total** | **106** | **3,4** | **-** | **1192** | **37,7** | **-** |
| *Type of School* | | | | | | |
| Public | 43 | 40,57 | **<0,0001** | 572 | 47,99 | **<0,0001** |
| Private | 63 | 59,43 |  | 620 | 52,01 |  |
| *Per capita income* | | | | | | |
| Lowest tercile | 42 | 39,62 | **<0,0001** | 143 | 12,00 | **<0,0001** |
| Middle tercile | 14 | 13,21 |  | 321 | 26,93 |  |
| Highest tercile | 50 | 47,17 |  | 728 | 61,07 |  |
| *Segregation Index* | | | | | | |
| Low (<0) | 59 | 55,66 | **<0,0001** | 738 | 61,91 | **<0,0001** |
| Medium (0 to 1,96) | 27 | 25,47 |  | 384 | 32,21 |  |
| High (≤1,96) | 20 | 18,87 |  | 70 | 5,87 |  |
| *Deprivation Index* | | | | | | |
| High Risk | 29 | 27,36 | <0,0001 | 387 | 32,47 | **<0,0001** |
| Medium Risk | 41 | 38,68 |  | 649 | 54,45 |  |
| Low Risk | 36 | 33,96 |  | 156 | 13,09 |  |
